# Role of *Trichoderma harzianum* in mitigating NaCl stress in Indian mustard (*Brassica juncea* L) through antioxidative defense system

**DOI:** 10.3389/fpls.2015.00868

**Published:** 2015-10-14

**Authors:** Parvaiz Ahmad, Abeer Hashem, Elsayed Fathi Abd-Allah, A. A. Alqarawi, Riffat John, Dilfuza Egamberdieva, Salih Gucel

**Affiliations:** ^1^Department of Botany, Sri Pratap CollegeSrinagar, India; ^2^Department of Botany and Microbiology, Faculty of Science, King Saud UniversityRiyadh, Saudi Arabia; ^3^Mycology and Plant Disease Survey Department, Plant Pathology Research Institute, Agriculture Research CenterGiza, Egypt; ^4^Department of Plant Production, Faculty of Food and Agricultural Sciences, King Saud UniversityRiyadh, Saudi Arabia; ^5^Department of Botany, University of KashmirSrinagar, India; ^6^Institute for Landscape Biogeochemistry, Leibniz Centre for Agricultural Landscape ResearchMüncheberg, Germany; ^7^Centre for Environmental Research, Near East UniversityNicosia, Cyprus

**Keywords:** NaCl, *Trichoderma harzianum* (TH), mustard, growth, osmolytes, H_2_O_2_, lipid peroxidation, antioxidants

## Abstract

Salinity stress affected crop production of more than 20% of irrigated land globally. In the present study the effect of different concentrations of NaCl (0, 100, and 200 mM) on growth, physio-biochemical attributes, antioxidant enzymes, oil content, etc. in *Brassica juncea* and the protective role of *Trichoderma harzianum* (*TH*) was investigated. Salinity stress deteriorates growth, physio-biochemical attributes, that ultimately leads to decreased biomass yield in mustard seedlings. Higher concentration of NaCl (200 mM) decreased the plant height by 33.7%, root length by 29.7% and plant dry weight (DW) by 34.5%. On the other hand, supplementation of *TH* to NaCl treated mustard seedlings showed elevation by 13.8, 11.8, and 16.7% in shoot, root length and plant DW respectively as compared to plants treated with NaCl (200 mM) alone. Oil content was drastically affected by NaCl treatment; however, *TH* added plants showed enhanced oil percentage from 19.4 to 23.4% in the present study. NaCl also degenerate the pigment content and the maximum drop of 52.0% was recorded in Chl. ‘*a*’. Enhanced pigment content was observed by the application of *TH* to NaCl treated plants. Proline content showed increase by NaCl stress and maximum accumulation of 59.12% was recorded at 200 mM NaCl. Further enhancement to 70.37% in proline content was recorded by supplementation of *TH*. NaCl stress (200 mM) affirms the increase in H_2_O_2_ by 69.5% and MDA by 36.5%, but reduction in the accumulation is recorded by addition of *TH* to mustard seedlings. 200 mM NaCl elevated SOD, POD, APX, GR, GST, GPX, GSH, and GSSG in the present study. Further enhancement was observed by the application of *TH* to the NaCl fed seedlings. NaCl stress suppresses the uptake of important elements in both roots and shoots, however, addition of *TH* restored the elemental uptake in the present study. Mustard seedlings treated with NaCl and *TH* showed restricted Na uptake as compared to seedlings treated with NaCl alone. In conclusion, *TH* proved to be very beneficial in imparting resistance to the mustard plants against NaCl stress through improved uptake of essential elements, modulation of osmolytes and antioxidants.

## Introduction

The farming land is declining gradually and the main reasons are, intensive use of agricultural practices, urbanization, biotic and abiotic stress etc. Among abiotic stresses the salinity problem is increasing at an alarming rate throughout the world. Salinity is responsible for the loss of crop production worth billions of dollars every year. It has been reported that about 7% of the total land on earth and 20% of the total arable area are affected by high salt content (Munns and Tester, 2008; Cabot et al., 2014). Salinity stress induces osmotic and ionic stress that leads to retarded growth in terms of both shoot and root length, fresh and dry weight (DW), reduced pigment content and hampers uptake of mineral elements (Ahmad et al., 2012, 2014). Sodium accumulation disturbs all physiological and biochemical processes including photosynthesis, respiration, membrane functions etc. The chlorophyll content dwindles with the increase in NaCl stress in chickpea (Rasool et al., 2013) and faba bean (Hashem et al., 2014). Accumulation of Na^+^ in the soil decreases the porosity, soil aeration and water conductance (Porcel et al., 2012). Mineral uptake by the plants is drastically hampered by the NaCl stress (Porcel et al., 2012), which directly affect growth biomass and yield of the plant. A prolonged salinity stress is responsible for secondary stress, i.e.; oxidative stress that generates reactive oxygen species (ROS) deleterious to biomolecules like, proteins, nucleic acids (DNA/RNA), membrane lipids etc. (Ahmad et al., 2010a,b; Ahmad, 2013). Plant cells generate ROS even under normal conditions but they are balanced by the scavenging system of the cell. When generation of ROS exceeds its quenching capacity, oxidative stress appears. Polyunsaturated fatty acids (PUFA) are more vulnerable to ROS attack and this leads to lipid peroxidation (Sánchez-Rodríguez et al., 2010). However, plants have protective mechanisms like enzymatic and non-enzymatic antioxidants against these ROSs. The enzymatic antioxidants are superoxide dismutase (SOD), ascorbate peroxidase (APX), catalase (CAT), glutathione reductase (GR) and non-enzymatic are ascorbic acid (ASA), glutathione (GSH) (Mittler, 2002; Ahmad and Sharma, 2008, 2010).

Besides the classic mycorrhizal fungi, rhizobia, and plant-growth-promoting rhizobacteria, endophytic fungi are reported to improve plant growth during stress (Egamberdieva et al., 2011, 2013; Berg et al., 2013; Hameed et al., 2014). *Trichoderma* sp. are endophytic plant symbionts widely used as biofertilizers for plant growth stimulation and as biocontrol agents for plant diseases (Brotman et al., 2010; Harman, 2011). *Trichoderma* strains are able to enhance plant tolerance to biotic and abiotic stresses such as drought and salinity (Mastouri et al., 2010; Shoresh et al., 2010), through enhanced root growth, nutritional uptake and by inducing protection against oxidative damage. Mastouri et al. (2012) reported that enhanced resistance of colonized water deficit plants by *Trichoderma harzianum* (*TH*) T22 is explained partly due to higher capacity to scavenge ROS and recycle oxidized ascorbate and glutathione, a mechanism that is expected to enhance tolerance to abiotic stresses. However, specific knowledge of mechanisms used by *TH* controlling multiple plant stress factors is still lacking and needs to be studied.

*Brassica juncea* L. (Czern and Coss) is commonly known as Indian mustard belongs to *Brassicaceae* family and is a multipurpose plant. The main constituent of the plant is mustard oil, which is well known in India and other countries for its edible property and medicinal importance as well. The residues of the plant can be used as biofuels, cattle feed and fertilizer for the soil (Jham et al., 2009). The mustard plant often experiences abiotic stress especially NaCl stress in arid and semi-arid regions of the world. India, inspite of being largest producer of edible oil faces shortage to meet even the daily requirements of its people. NaCl imposes hyperionic and hyperosmotic stress that interfers with the growth, biomass yield, and physio-biochemical attributes of mustard seedlings. Shrinking of cultivable land and exponential increase of human race are the two major concerns before the plant biologists. They need to look forward to make strategies to increase the crop production with the available land. One such sustainable strategy is the use of beneficial microbes in agriculture. *Trichoderma* species are used extensively to alleviate abiotic stress in variety of crop plants because of its high success rates. It has been observed that different hosts under stress conditions respond to beneficial micobes in a different manner. *B. juncea*, chosen as a model plant system for this study, is a major oil-yielding crop in India. Notably, *B. juncea* is susceptible to NaCl stress, which damages the crop production, hence oil yield. Use of beneficial microbes can be one of the sustainable strategies for improving *B. juncea* health under NaCl-salinity stress. Therefore, the present study was conducted to evaluate the effect of NaCl on growth, oil content, pigments and osmolytes and the mitigating role of *TH* in mustard seedlings. Production of ROS (H_2_O_2_) and during NaCl stress disrupts the membrane stability (lipid peroxidation) in mustard plants and positive role played by *TH* is too investigated. Protective nature of enzymatic and non-enzymatic antioxidants are also studied in mustard plants in presence and absence of NaCl and *TH*.

## Material and Methods

### Fungal Isolate

*Trichoderma harzianum* (T22) was obtained from culture collection of already infected maize plants and used for this study. This strain showed plant growth promotion of wheat seedlings and also alleviation of the adverse effects of salinity stress in wheat (Rawat et al., 2011). For preparation of fungal inocula, 3-5 disks of fresh cultured fungus grown on Potato Dextrose Agar (PDA) plates were inoculated in 100 ml of potato dextrose broth medium (PDB, DIFCO) in flasks and kept on a shaker for 5 days at 28°C. The mycelium obtained after incubation were lyophilized under vacuum. This lyophilized powder containing mycelium was mixed with talc powder and carboxy methyl cellulose (1.0%) and the final concentration of the carrier material per gram was 2 × 10^9^ cfu. *TH* was given to the pots at the rate of 10 g kg^-1^ soil before sowing. Pots without *TH* were treated as control.

### Plant Material

*Brassica juncea* L. (Czern and Coss) cv. Varuna, seeds were surface sterilized with sodium hypochlorite (0.5%, v/v) for 3 min, washed thoroughly with distilled water then germinated in a sterile Petri dish with 1% water agar in the dark at 28°C for 3 days. The sterility of seeds was tested on Nutrient agar and PDA by incubating plates for 3 days at 28°C.

### Pot Experiment

The soil used for the experiment has the following properties (%): sand (84.3); clay (8.2); silt (7.5); organic carbon, 0.17; total nitrogen, 0.007; (EC) = 7.12 dS/m; and pH 7.8. The soil was autoclaved for 40 min at 121°C (at 15 psi pressure), cooled down and then divided among plastic pots (300 g). After that equally germinated seeds were selected for sowing.

The seedlings were allowed to grow for an additional 3 weeks at average day/night temperatures of 28°C/15°C. After this different concentrations of NaCl (0, 100, 200 mM) were applied to the pots through Hoagland solution. TH is also applied to the soil in pots. Pots with out NaCl and TH served as control.

To maintain the moisture content of the pot, 100 ml of Hoagland nutrient solution along with dissolved NaCl was applied every alternate day to each pot except control, which received only nutrient solution. The experiment was laid out in a completely randomized design with five replicates. The plant leaves were collected for analysis after 45 days after treatment (DAT). The chemicals were procured from Sigma–Aldrich, Merck, and SRL. All chemicals were obtained in highest purity and were available commercially.

### Determinations

#### Growth Traits

The shoot and root length was measured manually by scale (100 cm), whereas DW was determined by drying the plant samples at 65°C for 72 h and then weighed.

#### Estimation of Oil Content

Solvent extraction method was employed for the estimation of oil content in mustard seedlings. Seeds (3 g) were ground in Na_2_SO_4_, the powder was kept in test tubes and hexane (20 ml) was added as mobile phase. The elution, which contains oil, was kept in a vile and was placed in hot water bath to evaporate the hexane. The oil in the vile was weighed and was calculated by the following formula:

Oil percentage = oil content/seed weight × 100.

#### Estimation of Pigments

Chlorophyll content of the leaves was determined by the method proposed by Arnon (1949). The absorbance was read at 663, 645, and 480 nm against 80% acetone used as a blank.

#### Estimation of Proline Content

For the estimation of proline, the procedure of Bates et al. (1973) was followed. Optical density (OD) was measured at 520 nm by spectrophotometer (Beckman 640 D, USA). The toluene was used as a blank.

#### Estimation of Hydrogen Peroxide and Lipid Peroxidation

The method of Velikova et al. (2000) was applied for the estimation of hydrogen peroxide. The optical density was measured at 390 nm by spectrophotometer (Beckman 640 D, USA).

Heath and Packer (1968) method was used for the determination of lipid peroxidation (amount of malondialdehyde produced) by thiobarbituric acid reaction. The OD was recorded at 600 nm and the blank used was 1% thiobarbituric acid (TBA) in 20% trichloroacetic acid (TCA). Extinction coefficient of 155 mM cm^-l^ was used for the calculation of malondialdehyde (MDA) concentration.

#### Extraction of the Enzymes

Leaf sample (10 g) were homogenized in 50 volumes of 100 mM Tris-HCl (pH 7.5) containing 5 mM DTT (Dithiothreitol), 10 mM MgCl_2_, 1 mM EDTA (Ethylenediaminetetraacetic acid), 5 mM magnesium acetate, 1.5% PVP-40 (Polyvinylpyrrolidone), 1 mM PMSF (phenylmethanesulfonyl fluoride) and 1 μg ml^-1^ aproptinin. After the filtration, the samples were centrifuged at 12,000 rpm for 10 min. The supernatant harvested was used as enzyme source. For the analysis of APX activity, tissues were separately homogenized with 2 mM AsA.

##### Enzyme assays

*Superoxide dismutase*. Estimation of SOD (EC 1.15.1.1) activity was executed following the photoreduction of nitrobluetetrazolium (NBT) (van Rossum et al., 1997). The OD was taken at 560 nm by spectrophotometer (Beckman 640 D, USA). SOD activity is inversely proportional to the NBT reduction. SOD unit is the amount of protein that restricts 50% photoreduction of NBT. SOD activity was expressed as enzyme unit (EU) mg^-1^ protein.

*Peroxidase*. Kar and Mishra (1976) method was followed for the estimation of peroxidase (POD) activity. The OD was taken at 420 nm by spectrophotometer (Beckman 640 D, USA). POD activity was expressed as change in EU mg^-1^ protein.

*Ascorbate peroxidase*. Ascorbate peroxidase activity was determined by following the method of Nakano and Asada (1981). The absorbance was read at 290 nm by spectrophotometer (Beckman 640 D, USA). EU mg^-1^ protein expresses the APX activity.

*Monodehydroascorbate reductase (MDHAR).* The method of Miyake and Asada (1992) was employed for the estimation of Monodehydroascorbate reductase (MDHAR, EC 1.6.5.4). MDAR activity was expressed as μmol NADPH oxidized/ (EU mg^-1^ protein).

*Dehydroascorbate reductase (DHAR)*. Dehydroascorbate reductase (EC: 1.8.5.1) activity was determined by the procedure of Nakano and Asada (1981). The absorbance was read at 265 nm for 1 min by spectrophotometer (Beckman 640 D, USA) using extinction coefficient of 14 mM^-1^ cm^-1^.

*Glutathione reductase*. For the determination of GR activity (EC 1.6.4.2), the method of Carlberg and Mannervik (1985) was followed. The absorbance showed decrease and was read for 2 min at 340 nm by spectrophotometer (Beckman 640 D, USA). The GR activity was calculated using the extinction co-efficient of NADPH of 6.2 mM^-1^ cm^-1^ and expressed as EU mg^-1^ protein.

*Glutathione *S*-sransferase (GST) and gaucol peroxidase (GPX) activity*. Activity of GST (EC: 2.5.1.18) was estimated according to the procedure of Hasanuzzaman and Fujita (2013). The rise in absorbance was read at 340 nm for 1 min by spectrophotometer (Beckman 640 D, USA). The GST activity was considered using the extinction coefficient of 9.6 mM^-1^ cm^-1^.

The method of Elia et al. (2003) was used to determine the GPX (EC: 1.11.1.9) activity. The absorbance was measured at 340 nm for 1 min by spectrophotometer (Beckman 640 D, USA). Extinction coefficient of 6.62 mM^-1^ cm^-1^ was used for the calculation of GPX activity.

*Catalase*. Catalase (EC 1.11.1.6) activity was determined by following the method of Luck (1974). The activity of CAT was calculated using the extinction co-efficient of 36 × 103 mM^-1^ cm^-1^ and expressed as EU mg^-1^ protein.

##### Extraction and measurement of ascorbate and glutathione

The method of Huang et al. (2005) was employed for the determination of ascorbate content. Fresh leaves (0.8 g) were crushed in 3 ml ice-cold acidic extraction buffer (5% meta-phosphoric acid containing 1 mM EDTA). The crushed material was subjected to centrifugation at 10,000 rpm for 20 min and the supernatant harvested were analyzed for ascorbate content.

The glutathione pool was assayed by the method of Yu et al. (2003) with some modifications described by Paradiso et al. (2008). Standard curves with known concentrations of GSH and GSSG were used. The content of GSH was calculated by subtracting GSSG from total GSH.

*Estimation of inorganic nutrients*. Dried shoot and root materials (100 mg) were powdered and digested in H_2_SO_4_/HNO_3_ mixture (1/5, v/v) for 24 h, then treated with HNO_3_/HClO_4_ mixture (5/1, v/v). Atomic absorption spectrophotometer (Analyst 300, PerkinElmer, Germany) was used for the measurement of elemental concentrations in the samples.

### Statistical Analysis

The statistical analysis (SPSS) was performed by one-way analysis of variance (ANOVA) followed by Duncan’s Multiple Range Test (DMRT). Each value is the mean ± SE for 5 replicates in each group. *P* ≤ 0.05 were considered as significant.

## Results

### *Trichoderma* Promotes Growth and Biomass Yield in NaCl Stressed Mustard Seedlings

The results related to the effect of NaCl stress in presence and absence of *TH* on growth and biomass yield in *B. juncea* is presented in **Table 1**. NaCl stress declined the growth in terms of length of shoot and root. The highest reduction in plant height was found to be 33.79% at 200 mM NaCl stress. Application of *TH* restored the plant height and increase of 14.88 and 13.81% was observed at 100 mM + *TH* and 200 mM + *TH* treatments respectively over NaCl treated plants.

**Table 1:** Effect on plant height (cm), root length (cm) and dry weight (g plant^-1^) under NaCl stress in presence and absence of *Trichoderma harzianum* (*TH*) in *Brassica juncea* seedlings.

Treatments	Plant height (cm)	Root length (cm)	Dry weight (g plant^-1^)
C	51.72 ± 1.57^a^	21.11 ± 1.00^a^	15.39 ± 0.88^a^
C + *TH*	53.19 ± 1.59^a^	23.72 ± 1.04^a^	16.61 ± 0.95^b^
100 mM	40.19 ± 1.31^b^	15.29 ± 0.94^b^	12.87 ± 0.73^c^
100 mM + *TH*	47.22 ± 1.42^c^	20.04 ± 0.98^c^	15.73 ± 0.90^a^
200 mM	34.24 ± 1.11^d^	11.82 ± 0.86^d^	10.08 ± 0.64^d^
200 mM + *TH*	39.73 ± 1.22^e^	14.81 ± 0.90^e^	12.11 ± 0.70^c^

*Values are means ± S.E (*n* = 5), superscript letters indicate significant difference between means at *p <* 0.05.*

Root length decreases by 27.56% at 100 mM and 48.74% at 200 mM NaCl treatments. However, co-application of *TH* mitigated the adverse effects of NaCl on root length. Length of root increases by 23.70 and 20.18% at 100 mM + *TH* and 200 mM+*TH* concentrations respectively as compared to plants treated with NaCl alone (**Table 1**).

Plant DW was severely affected by NaCl stress in present study. The maximum reduction of 34.50% was observed at 200 mM NaCl concentration relative to control. However, when *TH* was co-applied with NaCl, the DW increases by 18.18% and 16.76% at 100 mM + *TH* and 200 mM + *TH* concentrations respectively compared to plants treated with NaCl alone. The data clearly indicates that application of *TH* helped the mustard seedlings in restoring growth and biomass yield under NaCl stress.

### *Trichoderma* Improved the Oil Production in Mustard Plants Under NaCl Stress

**FIGURE 1:**
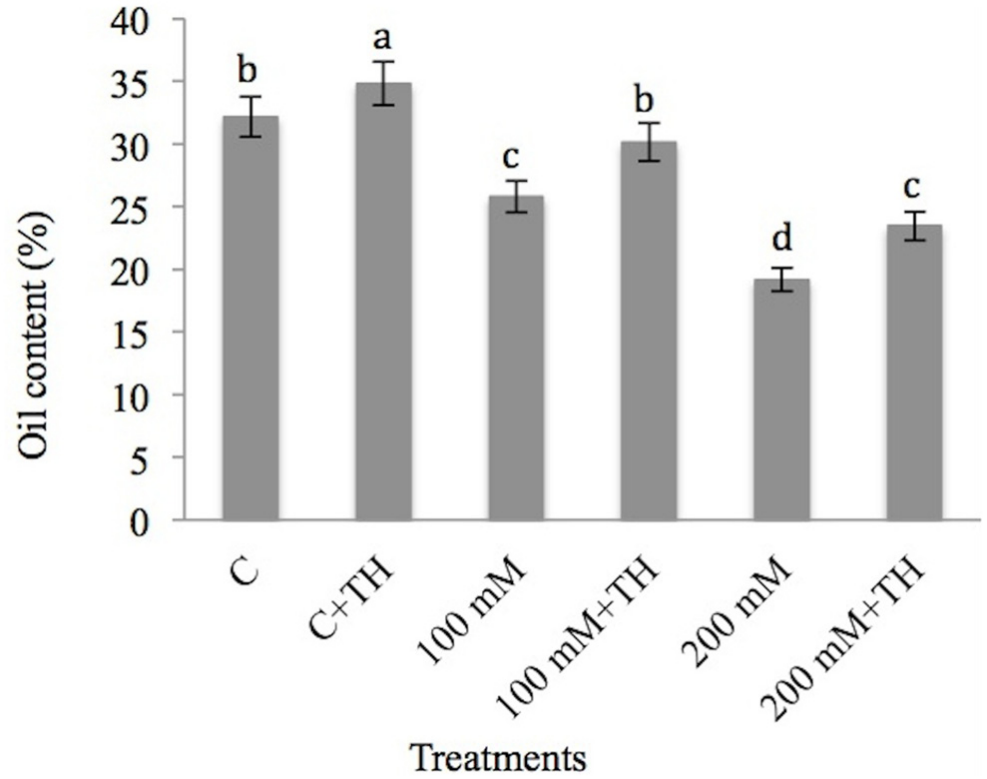
**Effect on oil content (%) under NaCl stress in presence and absence of *Trichoderma harzianum* (*TH*) in *Brassica juncea* seedlings.** Different letters indicate significant difference between means at *p < 0.05* (DMRT). Values are means ± SE (*n* = 5).

The results pertaining to the effect of NaCl and *TH* on oil content percentage is presented in **Figure 1**. NaCl treatment reduced the oil content by 25.77 and 19.35% at 100 and 200 mM NaCl treatments respectively relative to control. However, supplementation of *TH* improves the oil content by 30.12% at 100 mM + *TH* and 23.44% at 200 mM + *TH* treatment over the control. Increase in oil content percentage by *TH* proved its defensive nature to mustard seedlings against NaCl stress.

### Restoration of Pigment Content by *Trichoderma* in Mustard Seedlings Under NaCl Stress

The results related to the effect of NaCl and *Trichoderma* on pigment content is depicted in **Table 2**. NaCl stress decreases the pigment content and the maximum decrease of 52.00, 25.26, 42.59, and 28.57% in chl ‘*a*’ chl ‘*b*’, total chl and carotenoids respectively was observed at 200 mM NaCl concentration over the control. However application of *TH* to NaCl treated seedlings restored the pigment content. An increase by 15.15% in chl ‘*a*’, 12.34% in chl ‘*b*’, 13.88% in total chl and 14.63% in carotenoids was recorded at 200 mM + *TH* treatment as compared to 200 NaCl treatments alone. The restoration of pigments dipicts the positive role of *TH* in mustard seedlings under NaCl stress.

**Table 2:** Effect on chl *a, b*, total chlorophyll (mg g^-1^ FW), carotenoid (mg g^-1^ fw) and proline (μg g^-1^ fw under NaCl stress in presence and absence of *TH* in *B. juncea* seedlings.

Treatments	Chlorophyll a (mg g^-1^ fresh weight)	Chlorophyll b (mg g^-1^ fresh weight)	Total Chlorophyll (mg g^-1^ fresh weight)	Carotenoid (mg g^-1^ fresh weight)	Proline (μg g^-1^ FW)
C	1.75 ± 0.35^a^	0.95 ± 0.09^a^	2.70 ± 0.57^a^	0.49 ± 0.04^a^	56 ± 1.69^a^
C + *TH*	1.82 ± 0.39^b^	1.07 ± 0.12^b^	2.89 ± 0.61^b^	0.55 ± 0.06^b^	59 ± 1.71^a^
100 mM	1.13 ± 0.23^c^	0.82 ± 0.07^c^	1.95 ± 0.41^c^	0.41 ± 0.02^c^	97 ± 3.55^b^
100 mM + *TH*	1.38 ± 0.29^d^	0.93 ± 0.08^d^	2.31 ± 0.46^d^	0.48 ± 0.04^a^	128 ± 3.72^c^
200 mM	0.84 ± 0.06^e^	0.71 ± 0.04^e^	1.55 ± 0.32^e^	0.35 ± 0.01^d^	135 ± 4.06^d^
200 mM + *TH*	0.99 ± 0.10^f^	0.81 ± 0.06^f^	1.80 ± 0.36^f^	0.41 ± 0.02^d^	189 ± 4.21^e^

*Values are means ± SE (*n* = 5), different letters indicate significant difference between means at *p* < 0.05.*

### Effect of *Trichoderma* on Proline Content in Mustard Seedlings Under NaCl Stress

As for proline (**Table 2**), it increases by 42.26% and 59.12% at 100 and 200 mM NaCl treatments respectively in comparision to control. Addition of *TH* further increases the proline content by 56.25% at 100 mM + *TH* and 70.37% at 200 mM + *TH* treatments over the control. Increased accumulation of proline by *TH* application proves its protective nature against NaCl stressed mustard seedlings.

### *Trichoderma* Reduces Hydrogen Peroxide and Lipid Peroxidation in Mustard Seedlings Under NaCl

**FIGURE 2:**
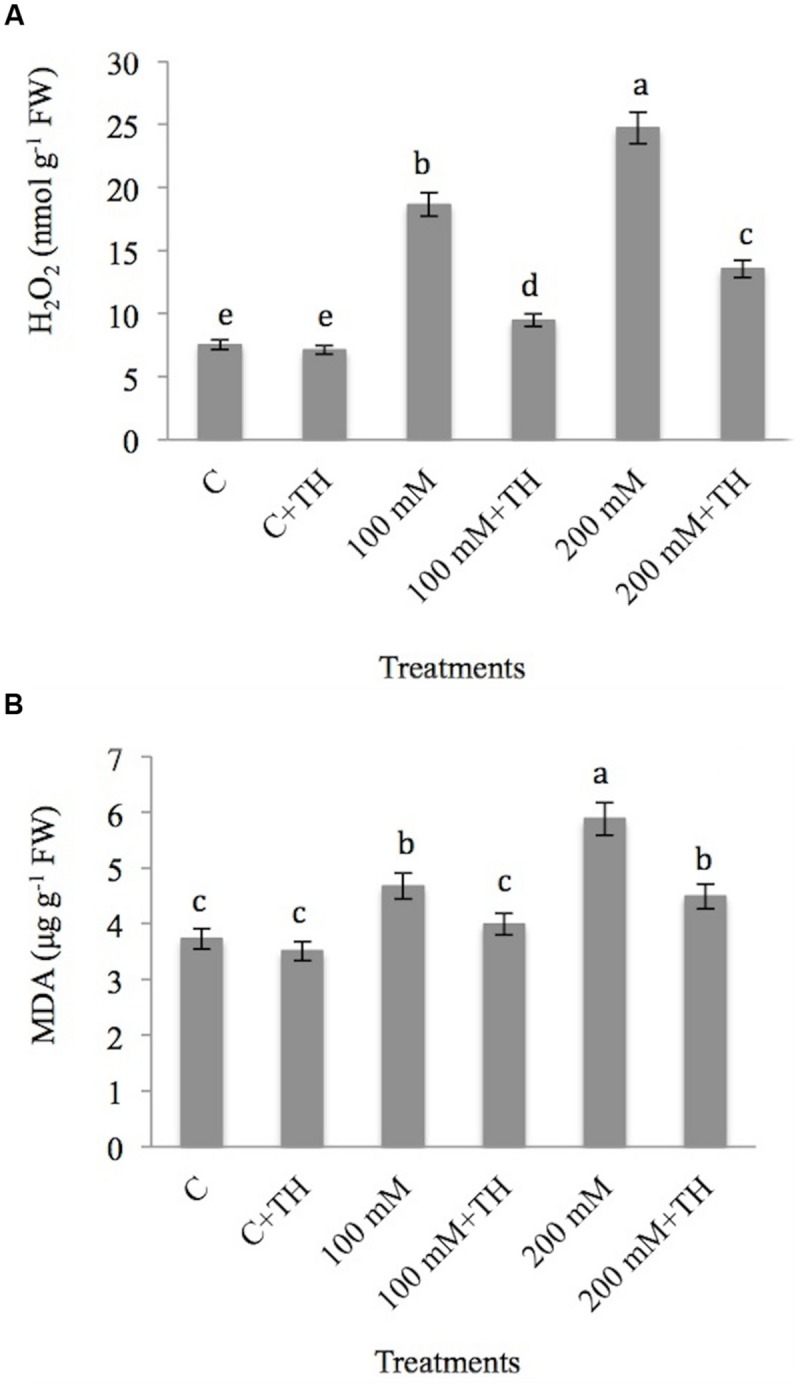
**Effect on **(A)** hydrogen peroxide (H_2_O_2_) and **(B)** malondialdehyde content (MDA) under NaCl stress in presence and absence of *TH* in *B. juncea* seedlings.** Different letters indicate significant difference between means at *p < 0.05*. Values are means ± SE (*n* = 5).

The results pertaining to the effect of NaCl and *TH* on hydrogen peroxide (H_2_O_2_) and MDA is depicted in **Figures 2A,B**. NaCl induces H_2_O_2_ by 59.67% at 100 mM and 69.57% at 200 mM NaCl concentration over the control. However, plants treated with *TH* in combination with NaCl showed less accumulation of 20.59% at 100 mM + *TH* and 44.50% at 200 mM + *TH* in H_2_O_2_ as compared with that of control.

Malondialdehyde, showed maximum rise of 36.56% at 200 mM and minimum of 20.12% at 100 mM NaCl concentration verses control. Decline in MDA content was observed in plants treated with *TH*. A decrease of 14.56 and 23.63% was observed at 100 mM + *TH* and 200 mM + *TH* respectively in MDA content as compared to 100 and 200 mM NaCl concentration (**Figure 2B**). From the above results it is concluded that *TH* plays a protective role as it mitigates the effect of H_2_O_2_ on lipid peroxidation.

#### Antioxidants

##### Superoxide dismutase and peroxidase

**FIGURE 3:**
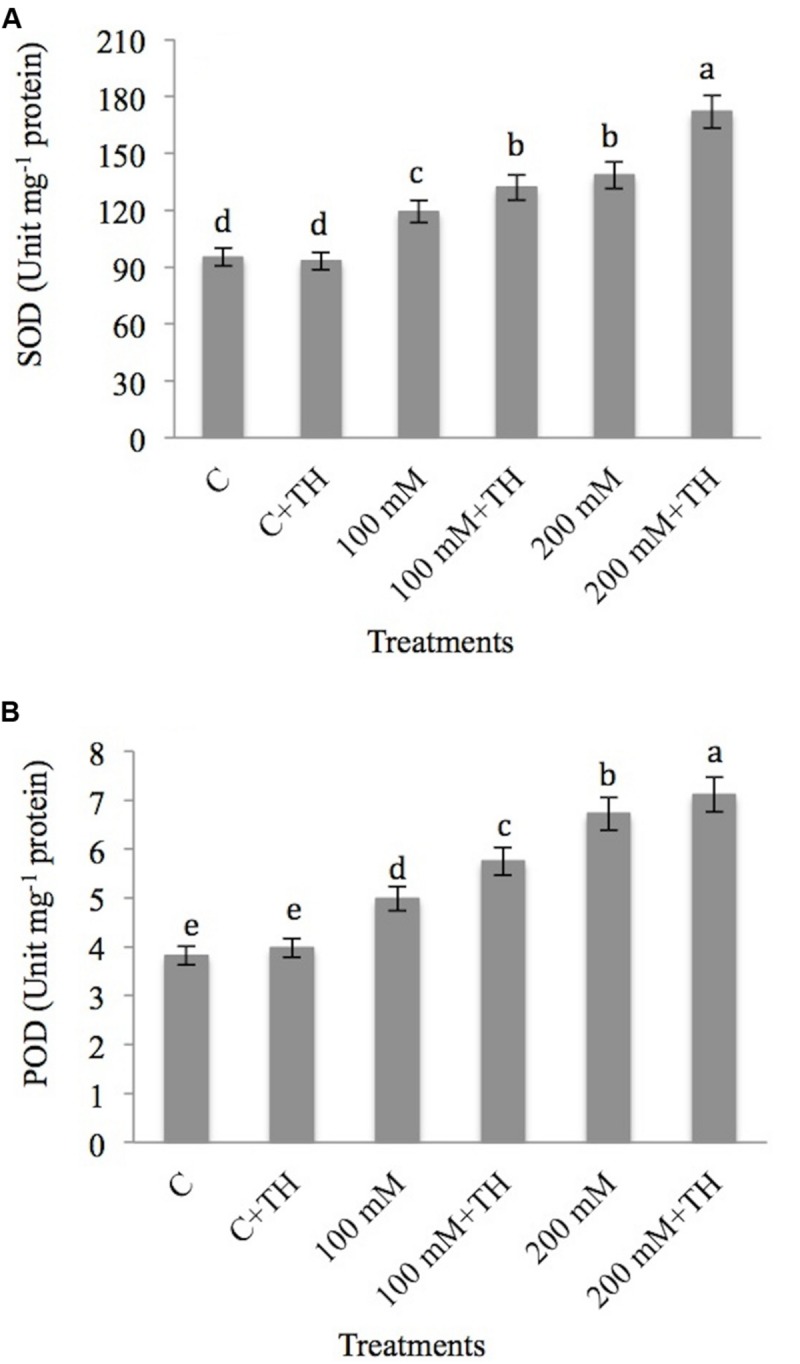
**Effect on **(A)** superoxide dismutase (SOD) and **(B)** peroxidase activity (POD) under NaCl stress in presence and absence of *TH* in *B. juncea* seedlings.** Different letters indicate significant difference between means at *p < 0.05*. Values are means ± SE (*n* = 5).

Superoxide dismutase increases with the increasing concentration of NaCl and the results are depicted in **Figure 3A**. SOD activity increased by 20.14 and 31.17% at 100 and 200 mM NaCl concentrations respectively as compared to control. Application of *TH* further elevated the SOD activity by 27.82% at 100 mM + *TH* and 44.61% at 200 mM + *TH* treatments over the control.

Regarding POD activity, minimum hike of 23.44% and maximum of 43.15% was recorded at 100 and 200 mM NaCl concentration respectively over the control plants. However, supplementation of *TH* to NaCl treated plants further enhanced the POD activity by 33.56% at 100 mM + *TH* and 46.27% at 200 mM + *TH* as compared to control (**Figure 3B**). Increasing activity of SOD and POD by the application of *TH* specify the defensive nature of *TH* on mustard seedlings under NaCl stress.

##### Ascorbate peroxidase, monodehydroascorbate reductase, dehydroascorbate reductase, glutathione reductase

**FIGURE 4:**
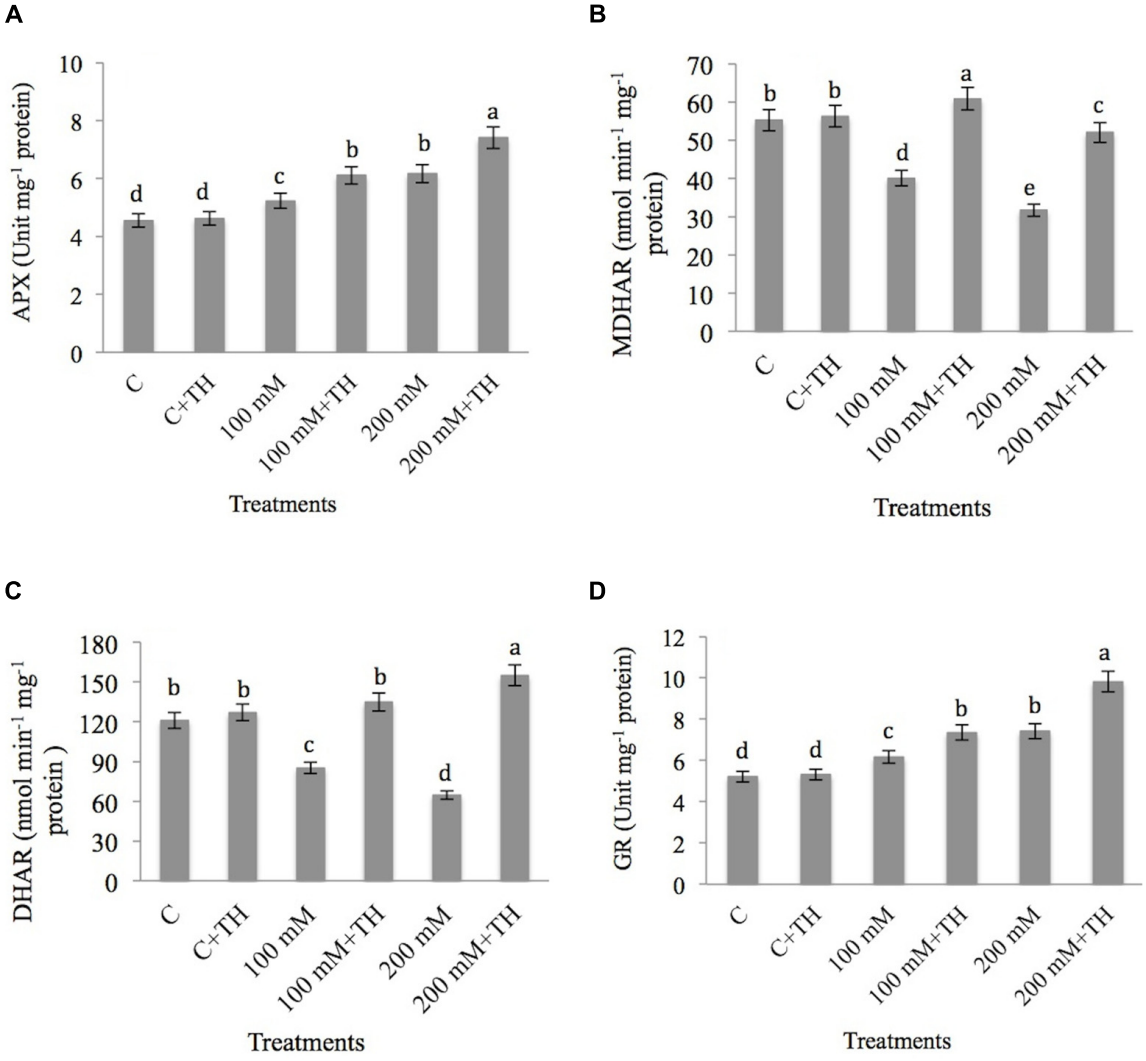
**Effect on **(A)** ascorbate peroxidase (APX), **(B)** monodehydroascorbate reductase (MDHAR), **(C)** dehydroascorbate reductase (DHAR), and **(D)** glutathione reductase (GR) under NaCl stress in presence and absence of *TH* in *B. juncea* seedlings.** Different letters indicate significant difference between means at *p < 0.05*. Values are means ± SE (*n* = 5).

The results related to the effect of NaCl and *TH* on APX activity in mustard seedlings is presented in **Figure 4A**. NaCl concentration increased the APX activity by 13.00% at 100 mM and 26.25% at 200 mM NaCl concentrations relative to control. Co-inoculation of *TH* to NaCl treated plants showed further increase of 25.53 and 38.59% in APX activity at 100 mM + *TH* and 200 mM + *TH* treatments respectively as compared to control.

NaCl decreases the activity of MDHAR by 27.42 and 42.60% at 100 and 200 mM NaCl concentratiosn respectively in comparison to control plants. However, addition of *TH* to NaCl treated plants increased the activity of MDHAR by 34.15% at 100 mM + *TH* as compared to 100 mM NaCl treatment. 200 mM + *TH* treatment also showed increase of 39.08% in MDHAR as compared to NaCl stress alone (**Figure 4B**).

As for DHAR (**Figure 4C**), NaCl stress decreased DHAR maximum by 46.51% at 200 mM NaCl concentration over the control. However, supplementation of *TH* to NaCl stressed plants increases the DHAR activity by 36.88% at 100 mM + *TH* and 58.24% at 200 mM + *TH* as compared to NaCl treated plants alone.

Glutathione reductase activity showed minimum rise of 15.55% and maximum of 29.78% at 100 and 200 mM NaCl respectively relative to control (**Figure 4D**). NaCl treated plants, inoculated with *TH* showed further increase in GR activity by 29.11% at 100 mM + *TH* and 46.94% at 200 mM + *TH* treatments over the control. *TH* maintains the activity of above antioxidant enzymes, which suggests its role in NaCl stress tolerance in mustard seedlings.

##### Glutathione *S*-transferase, guaiacol peroxidase, catalase

**FIGURE 5:**
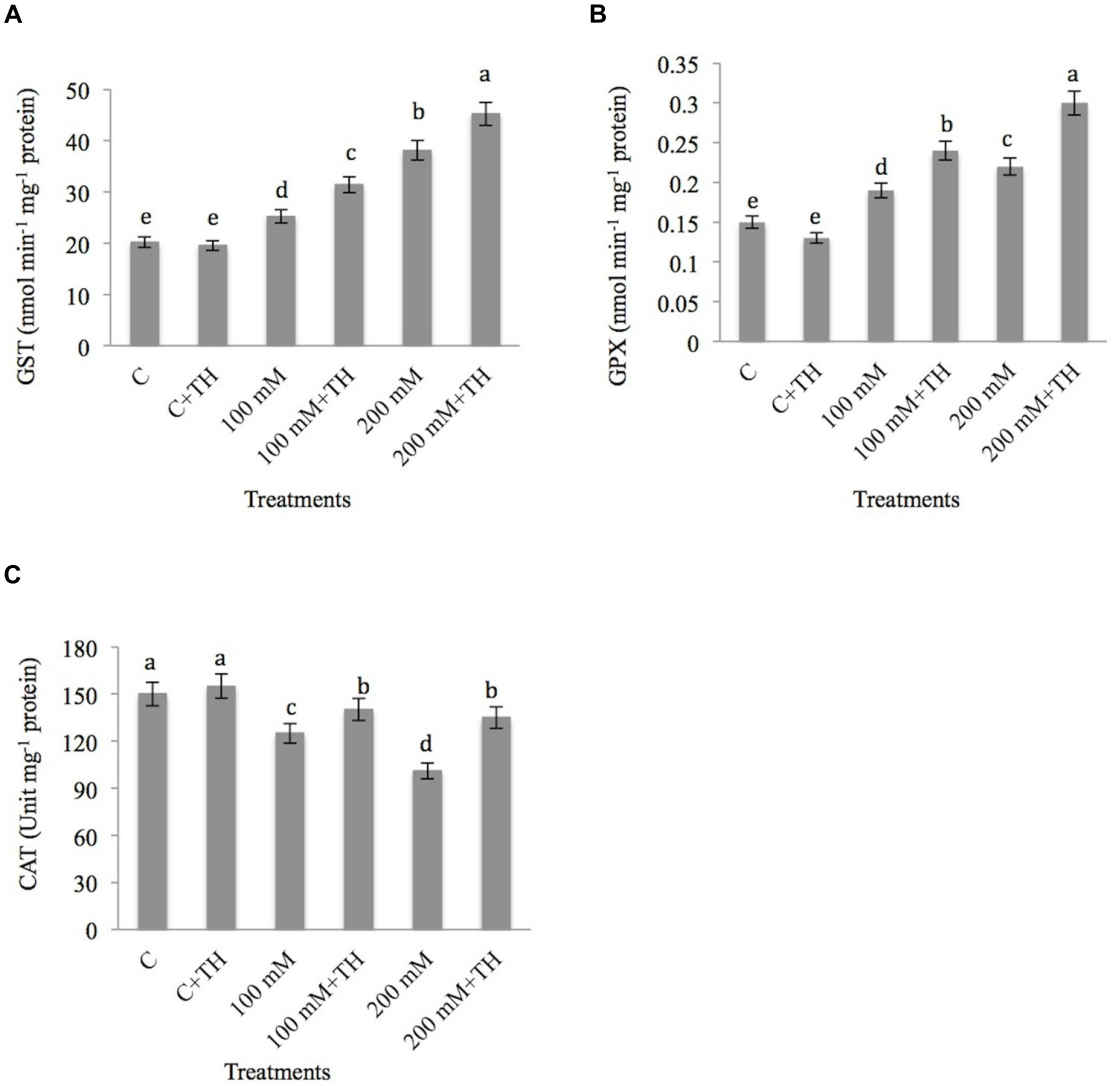
**Effect on **(A)** glutathione *S*-transferase (GST), **(B)** gaucol peroxidase (GPX), and **(C)** catalase (CAT) activity under NaCl stress in presence and absence of *TH* in *B. juncea* seedlings.** Different letters indicate significant difference between means at *p < 0.05* (DMRT). Values are means ± SE (*n* = 5).

The results related to the effect of NaCl and *TH* on GST is depicted in **Figure 5A**. GST increases with increased concentration of NaCl and the maximum elevation by 47.07% was recorded at 200 mM NaCl treatment. Minimum hike of 20.08% in GST was observed at 100 mM NaCl treatment. However, supplementation of *TH* to salt stressed plants further increased the GST activity by 35.80 and 55.44% at 100 mM + *TH* and 200 mM + *TH* treatments respectively as compared with control plants.

Regarding the GPX activity (**Figure 5B**), NaCl stress elevated the GPX activity by 21.05 and 31.81% at 100 and 200 mM NaCl stress respectively in comparision to control. Further enhancement of 37.50% at 100 mM + *TH* and 50.00% at 200 mM + *TH* treatments was observed when salt stressed plants were supplemented with *TH*.

Salt stress reduced the CAT activity by 16.66% at 100 mM and 32.66% at 200 mM concentrations as compared to control (**Figure 5C**). Addition of *TH* to salt stressed plants showed increase of 10.71 and 25.18% at 100 mM + *TH* and 200 mM + *TH* respectively in CAT activity as compared to NaCl treatments alone. The above results also recommend that *TH* conserve the activity of above antioxidants that showed their protective responsibility against NaCl stressed mustard seedlings.

##### Ascorbic acid, reduced glutathione, oxidized glutathione

**FIGURE 6:**
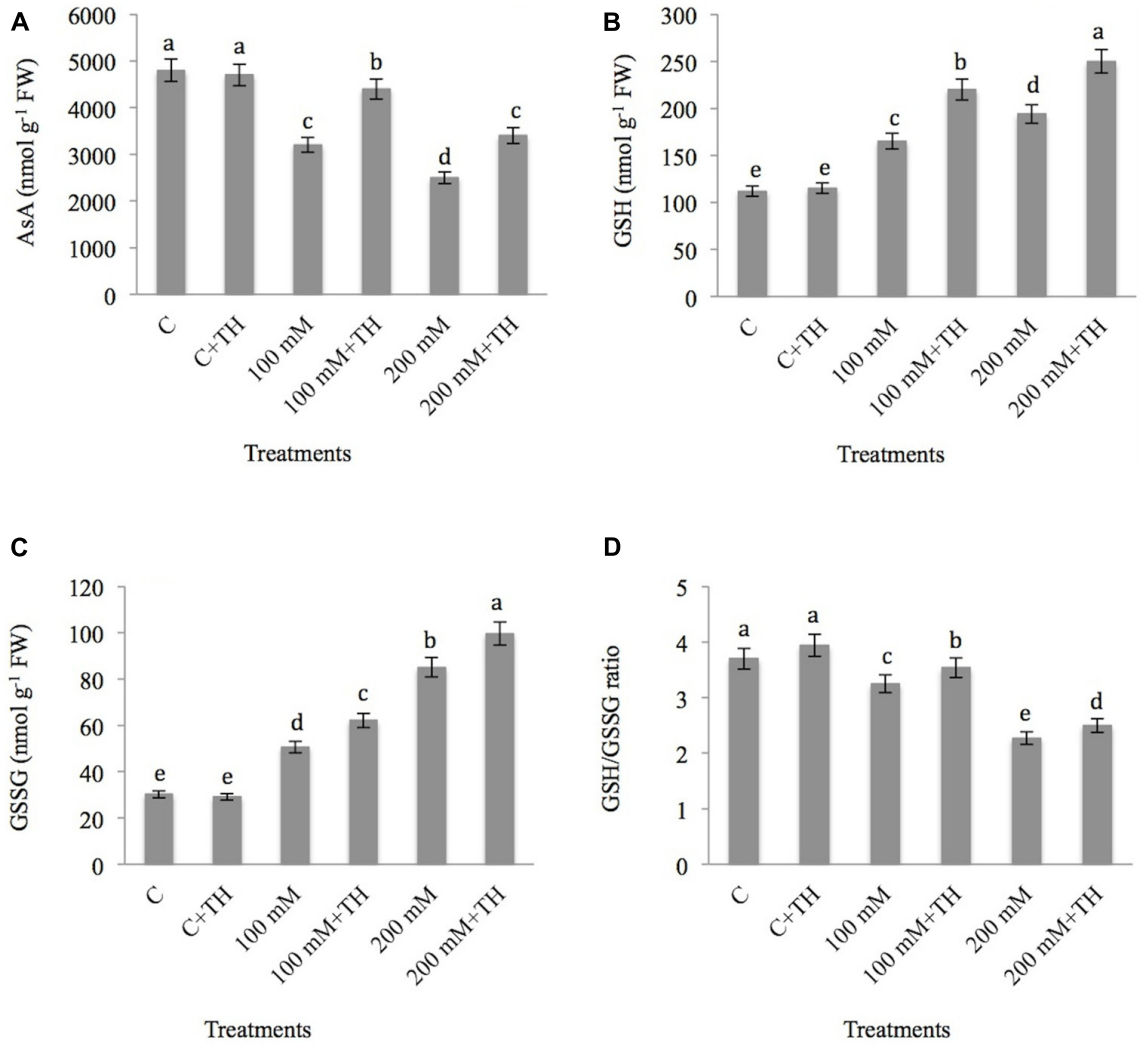
**Effect on **(A)** Ascorbic acid (nmol g^-1^ FW), **(B)** GSH (Reduced glutathione) (nmol g^-1^ FW), **(C)** GSSG (Oxidized glutathione) (nmol g^-1^ FW) and **(D)** GSH/GSSG ratio under NaCl stress in presence and absence of *TH* in *B. juncea* seedlings.** Different letters indicate significant difference between means at *p < 0.05* (DMRT). Values are means ± SE (*n* = 5).

The results related to the effect of NaCl and *TH* on AsA is presented in **Figure 6A**. AsA declines by 33.33 and 47.91% at 100 and 200 mM treatments respectively as compared with control. However, supplementation of *TH* to NaCl treated plants showed accumulation of 27.27% in AsA at 100 mM + *TH* as compared to 100 mM NaCl treatment. 200 mM + *TH* treatment also showed increase of 26.47% in AsA as compared to 200 mM NaCl concentration.

In the present study, GSH build up with increasing concentration of NaCl. The maximum enhancement of 42.26% was observed at 200 mM NaCl concentrations with respect to control (**Figure 6B**). Further improvement in GSH was observed in salt stressed plants supplemented with *TH*. An increase of 25.00 and 22.40% in GSH at 100 mM + *TH* and 200 mM + *TH* treatments respectively was observed as compared to NaCl stress alone.

Regarding GSSG (**Figure 6C**), NaCl stress increased the level of GSSG by 40.32% at 100 mM and 64.50% at 200 mM NaCl treatments over the control. Further accumulation was also observed; when salt treated plants were supplemented with *TH*. An elevation of 51.36 and 69.67% at 100 mM + *TH* and 200 mM + *TH* respectively in GSSG were observed in the present study as compared to control.

NaCl stress decreases the GSH/GSSG ratio by 12.16 and 38.64% at 100 and 200 mM NaCl concentrations respectively in comparision to control (**Figure 6D**). However, *TH* treatment to NaCl stressed plants showed improvement in GSH/GSSG ratio by 8.19% at 100 mM + *TH* and 9.20% at 200 mM + *TH* as compared to 100 and 200 mM NaCl treatments. The above data suggests that *TH* application preserve the non-enzymatic antioxidants, which provides tolerance to mustard seedlings under NaCl stress.

### Improved Uptake of Essential Elements by *Trichoderma* in NaCl Stressed Mustard Seedlings

NaCl treatments hamper the uptake of S, Mn, Mg, Ca, and K in both shoots and roots (**Table 3**). Maximum decrease by 25.94, 55.14, 29.86, 33.60, and 49.58% in S, Mn, Mg, Ca, and K respectively in shoots was observed at 200 mM NaCl concentration as compared to control. Roots also showed reduction by 45.56% in S, 24.42% in Mn, 21.52% in Mg, 21.89% in Ca and 28.64% in K at 200 mM NaCl treatments over the control. However, application of *TH* restores the uptake of above elements to appreciable level in both roots and shoots. Enhanced uptake of nutrients by *TH* in mustard seedlings signifies its role in tolerance against NaCl stress.

**Table 3:** Effect on uptake of minerals (μmol g^-1^ dry wt.) under NaCl stress in presence and absence of *TH* in *B. juncea* seedlings.

Mineral nutrition (μ mol g^-1^ dry wt.)	C	C + *TH*	100 mM	100 mM + *TH*	200 mM	200 mM + *TH*
Shoot S	127 ± 3.31^a^	132 ± 3.38^b^	115 ± 3.16^c^	123 ± 3.27^d^	94.05 ± 2.81^e^	103 ± 3.05^f^
Shoot Mn	26.91 ± 1.37^a^	29.21 ± 1.44^b^	14.11 ± 1.15^c^	19.23 ± 1.26^d^	12.07 ± 1.07^e^	16.11 ± 1.21^f^
Shoot Mg	298 ± 4.98^a^	315 ± 5.11^b^	241 ± 4.39^c^	269 ± 4.72^d^	209 ± 4.01^e^	224 ± 4.17^f^
Shoot Ca	125 ± 3.28^a^	131 ± 3.37^b^	95 ± 2.83^c^	112 ± 3.13^d^	83 ± 2.70^e^	97 ± 2.85^f^
Shoot K	716 ± 9.51^a^	725 ± 9.59^b^	548 ± 7.21^c^	615 ± 8.11^d^	361 ± 5.46^e^	421 ± 6.97^f^
Root S	395 ± 5.84^a^	415 ± 6.12^b^	297 ± 4.96^c^	334 ± 5.31^d^	215 ± 4.11^e^	240 ± 4.45^f^
Root Mn	94.11 ± 2.81^a^	99.10 ± 2.90^b^	83.09 ± 2.71^c^	92.15 ± 2.77^d^	71.12 ± 2.55^e^	79 ± 2.66^f^
Root Mg	971 ± 11.31^a^	996 ± 11.88^b^	815 ± 10.22^c^	895 ± 10.91^d^	762 ± 9.85^e^	798 ± 10.01^f^
Root Ca	201 ± 3.97^a^	215 ± 4.12^b^	179 ± 3.69^c^	197 ± 3.90^d^	157 ± 3.46^e^	170 ± 3.61^f^
Root K	1501 ± 13.01^a^	1570 ± 13.55^b^	1285 ± 11.85^c^	1430 ± 12.85^d^	1071 ± 10.21^e^	1190 ± 11.06^f^

*Values are means ± SE (*n* = 5), superscript letters indicate significant difference between means at *p* < 0.05.*

**Table 4** showed elevation in Na accumulation in both shoots and roots as the NaCl concentration goes up, but supplementation of *TH* decreased the aggregation of Na by 45.24 and 53.67% in shoots and roots respectively at 200 mM + *TH* treatments in comparision to NaCl treated seedlings alone. Restricted uptake of Na by *TH* signifies its protective nature against NaCl stress in mustard seedlings.

**Table 4:** Effect on Na uptake under NaCl stress in presence and absence of *TH* in shoots and roots of *B. juncea* seedlings.

Na uptake (mg g^-1^ DW)	C	C + *TH*	100 mM	100 mM + *TH*	200 mM	200 mM + *TH*
Shoot	ND	ND	8.39 ± 0.83^a^	4.16 ± 0.47^b^	13.77 ± 1.10^c^	7.54 ± 0.69^d^
Root	ND	ND	15.48 ± 1.25^a^	7.63 ± 0.71^b^	26.10 ± 1.57^c^	12.09 ± 1.06^d^

*Values are means ± SE (*n* = 5), different letters indicate significant difference between means at *p <* 0.05.*

## Discussion

*Trichoderma* has an important role in metabolic processes of host plants that could impart tolerance against NaCl stress. *Trichoderma* sp have the capacity to induce systemic resistance, increase nitrogen use efficiency (Harman et al., 2004; Shoresh et al., 2010), increases water holding capacity (Berg et al., 2013; Hameed et al., 2014), induces osmolytes to protect the plants from osmotic stress, help in uptake of essential minerals, enhances photosynthetic efficiency (Hashem et al., 2014) etc.

NaCl affects the plant growth and biomass yield and is reported by different workers (Azooz et al., 2011; Rasool et al., 2013; Ahmad et al., 2014). The present study also reported reduction in shoot height, root length, plant DW and oil content under NaCl stress. Application of *TH* in combination with NaCl mitigated the negative effect of NaCl and the results corroborates with the findings of Mastouri et al. (2012) in tomato. Contreras-Cornejo et al. (2009) also reported enhanced biomass production on supplementation of *Trichoderma* to *Arabidopsis*. Availability of *Trichoderma* to the rice roots significantly enhanced root length and biomass yield and may be attributed to several growth promoting mechanisms like (i) mineral availability, (ii) availability of phytohormones, (iii) release of elicitors etc. (Doni et al., 2014). Plant growth hormones like cytokinins-like molecules, e.g. zeatin and gibberellin GA_3_ or GA_3_ related are produced in *Trichoderma* inoculated plants and have the ability to enhance the growth and development of the plants even under salt stress (Iqbal and Ashraf, 2013; Rawat et al., 2013; Zhang et al., 2013).

Negative effect of NaCl stress is also correlated with the decrease in oil yield in the present study and the results are in accordance with the findings of Ashraf and Akhtar (2004) in *Foeniculum vulgare* Mill. Increasing concentrations of NaCl resulted in decline in essential oil content in different plants (Tabatabaie and Nazari, 2007; Aziz et al., 2008). Decrease in oil content due to NaCl stress may be due to the limited supply of cytokinin from root to shoot which resulted in variations in cytokinin: abscisic acid (ABA) ratio in leaf (El-keltawi and Croteau, 1987). Application of *TH* resored the oil content of mustard seedlings under NaCl stress in the present study. Beneficial microbes have been reported to decrease the accumulated ABA during NaCl stress and make the transportation of cytokinins from root to shoot easy (Aroca et al., 2013; Hashem et al., 2015).

According to the published literature, NaCl stress inhibits the photosynthetic pigments in majority of plant species. Indeed the data showed in **Table 2** indicated that Chl and carotenoid syntheses were negatively affected by NaCl stress. Decrease in Chl content might be due to (i) the inhibition of synthesis of important enzymes, such as δ-aminolevulinic acid dehydratase and protochlorophyllide reductase, which are involved in Chl biosynthesis and (ii) impairment in the supply of Mg^2+^, Fe^2+^, Zn^2+^, and Mn^2+^ that are required for the synthesis of Chl (Padmaja et al., 1990; Van Assche and Clijsters, 1990; Küpper et al., 1996). Carotenoids possess an antioxidant property and provides photo protection to chlorophylls by scavenging ROS (Behera et al., 2002). Thus decrease in carotenoid content by NaCl stress results in overproduction of ROS that subsequently hampers plant growth by inducing oxidative damage to DNA, RNA, and proteins (Mishra et al., 2006; Ahmad et al., 2010a,b). Application of *TH* has restored the chlorophyll and carotenoid content to appreciable level in the present study and the results corroborates with the findings of Rawat et al. (2011) and Zhang et al. (2013). *TH* increases the uptake of essential elements especially Mg^2+^ that was negatively affected by NaCl stress, hence the chlorophyll synthesis increases in *TH* inoculated plants. Another reason for increased pigment content in plants may be the production of phytohormones that contributes for the stimulation of chlorophyll content (Martínez-Medina et al., 2014; Resende et al., 2014). The increase in photosynthetic pigments by *TH* colonization in plants may also be due to inhibition of Na uptake (Iqbal and Ashraf, 2013).

Proline an important osmolyte maintains the cell osmoregulation under NaCl stress (Ahmad et al., 2010b; Rasool et al., 2013). Our data also reflects the increase in proline content in mustard plants under NaCl stress (**Table 2**). *TH* inoculated mustard plants showed further accumulation of proline in the present study. Evelin et al. (2009) reported that AM fungi colonized plants showed maximum accumulation of solutes that provides protection to the cell from NaCl stress. Salt treated *Arabidopsis* seedlings inoculated with *Trichoderma* sp. showed more accumulation of proline as compared to control seedlings (Contreras-Cornejo et al., 2014). Proline is reported to have antioxidant property that could scavenge the ROS and protects the cell from oxidative damage (Ahmad et al., 2010b; Jogaiah et al., 2013). Proline has also a leading role in energy storage (i.e., C and N) under NaCl stress (Aggarwal et al., 2012). Higher accumulation of proline increases the N fixation in plants (Hashem et al., 2015).

Salt stress is responsible for the generation of ROS like hydrogen peroxide in the cell (Azevedo Neto et al., 2005; Ahmad et al., 2010a,b). The increase in H_2_O_2_ in present study corroborates with the findings of Ashraf et al. (2010) in wheat. Giannakoula and Ilias (2013) also showed increased level in H_2_O_2_ in tomato on exposure to NaCl. H_2_O_2_ is the only ROS that can diffuse through aquaporins in the membranes and over larger distances within the cell (Bienert et al., 2007). However, plants treated with *TH* showed less accumulation of H_2_O_2_ in the present study that may be attributed to confer bioprotection against NaCl stress. Hajiboland et al. (2010) also reported that colonized tomato plants with AMF showed less accumulation of H_2_O_2_ so lower oxidative damage as compared to non-mycorrhizal plants. *Citrus* plants inoculated with *Glomus versiforme* or *G. mosseae* under salt stress showed lower levels of H_2_O_2_ concentration as compared to non-inoculated plants (Wu et al., 2010). Rawat et al. (2013) also observed the minimum level of H_2_O_2_ in *TH* treated chickpea plants, whereas significantly higher level of H_2_O_2_ was maintained in control plants under both saline and non-saline soil conditions. At cellular level these plants are better equipped with efficient free radical quenching system that offers protection against oxidative stress.

Lipid peroxidation is estimated through the accumulation of MDA and has been used as a promising criterion for determining the sensitivity of plants to saline stress (Ashraf et al., 2010; Ahmad et al., 2014). Increase in MDA content under salt stress is also reported in tomato (Li, 2009), mulberry (Ahmad et al., 2014), Okra (Saleem et al., 2011). In present study a decrease in MDA content was observed in plants treated with *TH*. Similar results observed for chickpea showed significantly higher accumulation of MDA in non-inoculated than inoculated *Trichoderma* plants (Rawat et al., 2013). Plants treated with AMF showed less accumulation of MDA content (Wu et al., 2010). AMF inoculated plants increase the antioxidant enzymes that scavenge these free radicals and minimizes the attack on lipid membranes, hence decreases lipid peroxidation. It has already been reported that *Trichoderma* induces phytohormones like salicylic acid (SA) and jasmonic acid (JA; Martínez-Medina et al., 2011). The intrinsic SA may reduce H_2_O_2_ content due to its role as an antioxidant in counteracting the generation of H_2_O_2_ to some extent under NaCl stress. Lipid peroxidation decreases due to accumulation of antioxidants that could scavenge the ROS and peroxidation of membranes is minimized. *Trichoderma* induced the expression of many antioxidant enzymes that directly or indirectly scavenges the ROS and minimizes the effect on plasma membrane. *Trichoderma* also induces the expression of stress related proteins like glutathione *S*-transferase (GST), glutathione dependent formaldehyde dehydrogenase, and POD, which could lower down the MDA content (Hashem et al., 2014).

Superoxide dismutase increases with increasing concentration of NaCl is also reported by various workers in chickpea (Rasool et al., 2013), in tomato (Abdel Latef and Chaoxing, 2011), in broad bean (Azooz et al., 2011) and in *Morus alba* (Ahmad et al., 2014).

NaCl induces the activity of peroxidase to protect the plants from damage. Peroxidase helps in conversion of H_2_O_2_ to water and oxygen. In present study *TH* treated plants showed significant increase in POD, as compared to non-inoculated plants. Gusain et al. (2014) showed that *Trichoderma* increased the SOD, and POD in rice cultivars provides tolerance to these plants under water stress. Inoculated plants with AMF showed higher activity of SOD than non-inoculated plants (Borde et al., 2012).

Ascorbate is vital antioxidant in ascorbate-glutathione cycle. *Trichoderma* treated plants have been observed to accumulate more ascorbate in its reduced form. The enzymatic unit associated with the regeneration of reduced ascorbate is MDHAR. MDHAR was shown to be crucial for the mutualistic interaction between *Arabidopsis* and *Piriformospora indica* (Vadassery et al., 2009). Interestingly, the gene responsible for expression of MDHAR is extreemly expressed in cucumber and *Arabidopsis* upon inoculation with *Trichoderma*. Pre-treatment with *Trichoderma* in *Arabidopsis* under salt stress showed induced expression of different transcripts having vital role in osmoregulation and oxidative stress management (Brotman et al., 2013). The procedure reveals that *T. asperelloides* can induce plant tolerance to salt stress (Brotman et al., 2013). Mastouri et al. (2012) also reported that pre- treatment of tomato seedlings with *TH* T22 enhances drought stress through the upregulation of antioxidant machinery. *Trichoderma* strains (*T. asperelloides* and *T. harzianum*) activated antioxidant machinery to recycle the oxidized ascorbate has been reported in different plants (*Arabidopsis*, cucumber, and tomato), so as to improve the tolerance mechanism to a wide array of abiotic stresses (Mastouri et al., 2012; Brotman et al., 2013).

*Trichoderma* induces changes in host plants and these changes are directly linked to stress related genes and proteins (Brotman et al., 2013). Inoculation of cucumber seedlings with *Trichoderma* showed induced expression of *MDAR, APX1*, and *GST* genes and impart induction of antioxidant machinery against NaCl induced oxidative stress. *MDAR* gene expression level was increased to 15 fold in the above study. Up-regulation of *sod(Mn)* and *sod(cu)* genes by *Trichoderma* have also been reported in cucumber under NaCl stress (Brotman et al., 2013).

*Trichoderma harzianum* increased the GR and GST activities under salt stress condition as compared to control. GR is a flavo-protein oxidoreductase, present in both prokaryotes as well as in eukaryotes (Romero-Puertas et al., 2006). It is a potential enzyme of the Ascorbate–Glutathione system. GR catalyzes the reduction of glutathione, which is associated with regulation of many plant metabolic and antioxidative processes. The main role of GR is to catalyze the NADPH dependent reaction of disulphide bond of GSSG and thus maintains the reduced pool of glutathione (Ansel et al., 2006).

Glutathione *S*-transferase can eliminate membrane lipid peroxides, products of oxidative DNA degredation etc. (Berhane et al., 1994). Roxas et al. (2000) demonstrated that transgenic tobacco seedlings overexpressing the GST gene showed enhanced growth under different stresses. Yu et al. (2003) also reported that overexpression of GST in transgenic rice plants showed enhanced tolerance to Cd stress.

*Trichoderma harzianum* treatment to NaCl stressed plants showed increase in GSH/GSSG ratio as compared to control plants. GSTs minimizes peroxides by involving GSH and provide the scavengers for cytotoxic and genotoxic compounds. GPXs use glutathione to reduce H_2_O_2_ and organic and lipid hydroperoxides, and thus helps to shield the cells from oxidative damage (Noctor et al., 2002). The decrease in the oxidative damage may be due to the high induction of stress-associated proteins like GST, glutathione dependent formaldehyde dehydrogenase (FALDH) and peroxidase. Harman et al. (2004) observed the similar results in maize by inoculating *Trichoderma* isolate T-22. During ROS production under environmental stress these detoxifying proteins induced by *Trichoderma* inoculation acts as quenching agents and protects the cell from oxidative damage (Rawat et al., 2011).

Catalase is an important antioxidant enzyme that directly dismutates hydrogen peroxide and is essential for detoxification of ROS under stress (Van Breusegem et al., 2001). In comparision to CAT and POD, APX has more affinity for H_2_O_2_, thus it may play a vital role in management or detoxification of ROS under stress.

Glutathione has been reported to react with range of ROS (Noctor et al., 2002) and is involved in reduction of H_2_O_2_ to water in ascorbate-GSH cycle (Noctor and Foyer, 1998). Herouart et al. (1993) also reported that GSH induces expression of Cu/Zn SOD in tobacco. It has been reported GSH dependent enzymes; GR and GST increases in *Trichoderma* treated plants (Bailey et al., 2006; Shoresh and Harman, 2008). The GR is involved in the maintainence of GSH/GSSG ratio, that is necessary for the regeneration of ascorbate and for the initiation of many important enzymes associated with CO_2_-fixation (Noctor and Foyer, 1998). The protective nature of glutathione transferase might be due to its role in elimination of 4-hydroxyalkenals (membrane lipid peroxide) and proponal (DNA degredation product) by conjugating them with GSH (Berhane et al., 1994). It has also been reported that glutathione transferase directly detoxify lipid peroxides (Cummins et al., 1999) because some glutathione transferase have glutathione peroxidase activity (Cummins et al., 1999).

NaCl stress has been found to hamper the uptake of mineral nutrients like, S, Mn, Mg, Ca, and K in the present study. The results corroborates with the findings of Talei et al. (2012) in *Andrographis paniculatai*. Patel et al. (2010) also reported reduced accumulation of K and Ca in cowpea under NaCl stress. Na ion competes with the K for binding sites thus hampers the uptake of K. Uptake of S, Mn, Mg, and Ca also got hampered due to NaCl stress and is also reported by Pandolfi et al. (2012) in pea, Kanwal et al. (2013) in wheat and Iqbal and Ashraf (2013) in wheat. TH application has restored the uptake of essential elements in mustard plants under NaCl stress. Kadian et al. (2013) reported that plants inoculated with AMF enhance K uptake under NaCl stress. Evelin et al. (2009) also reported the enhancement of K:Na ratio in roots and shoots of plants and assist the plant in maintaining the K mediated enzymatic process and protein synthesis. *TH* produces plant growth regulators (Zhang et al., 2013) like α-naphthaleneacetic acid (NAA), indole-3-acetic acid (IAA) and indole-3-butyric acid (IBA) that imparts significant modifications in biological reactions and may be a reason for alleviation of NaCl stress (Iqbal and Ashraf, 2013).

Accumulation of Na ions increases with rise in NaCl concentrations in the present study. Kao et al. (2006) reported that increase in Na accumulation in three genotypes of soybean under salt stress. Agglomeration of Na in salt tolernt and suceptible varieties of *Azolla* is reported by Masood et al. (2006). Maggio et al. (2007) also confirmed building up of Na ions with increased levels of NaCl in tomato. However, the endophytic inoculated plants showed decrease in Na accumulation in plants and the results are also confirmed by many authors (Talaat and Shawky, 2011; Evelin et al., 2012; Contreras-Cornejo et al., 2014). According to Giri et al. (2007) mycorrhizal treated plants maintains K/Na ratio. K is an important element and an osmotic regulator in the plant cell. Na competes with K in uptake because both elements have similar physico-chemical structure (Serrano and Rodriguez-Navarro, 2001). This is the main reason why NaCl increases Na accumulation and lowers K, thus disturbs K/Na ratio (Zhang et al., 2010). *TH* inoculated mustard seedlings clearly indicated that concentration of K increases and that of Na decreases. Improvement in growth in *TH* inoculated seedlings under NaCl stress has been partly correlated to decreased accumulation of Na (Giri et al., 2007; Hajiboland et al., 2010; Wu et al., 2013).

*Trichoderma* is responsible for release of several compounds that might help the plant to tolerate the harsh environmental conditions. The plants inoculated with *Trichoderma* strains showed an increased defense-and stress-related compounds like JA, SA, and ABA (Martínez-Medina et al., 2011; Rubio et al., 2014). It is reported that SA is involved in activation of antioxidants against different environmental stresses like heat, NaCl, UV, heavy metals etc. (Mishra and Choudhuri, 1999; Ahmad et al., 2011). It is also reported that *Trichoderma* strains apart from producing plant growth hormones, they also synthesize 1-aminocyclopropane-1-carboxylate (ACC) deaminase, which cleaves ACC, the immediate precursor of the phytohormone ethylene and confers ‘induced systemic tolerance’ (IST) against drought and salt stress in plants (Viterbo et al., 2010). Heterologous expression of ACC deaminase from *T. asperellum* improved the growth performance of *Arabidopsis thaliana* under normal and salt stress conditions (Zhang et al., 2015). The inoculation of *Trichoderma* sp. in plants increased deep roots, which helped in more water acquisition and uptake of nutrients and thereby increasing the plants ability to resist abiotic stresses (Azarmi et al., 2011).

## Conclusion

NaCl stress has been found to impose deleterious effects on mustard plants. The growth, biomass yield, oil content, pigment system was negatively affected especially at higher levels of NaCl stress. However, *TH* was found to mitigate the detrimental effects of NaCl stress in mustard seedlings. *TH* inoculated plants restored the pigment content, plant growth and development. The increase in proline content was found to be very useful in providing tolerance to these plants under NaCl stress. Both enzymatic (SOD, POD, CAT, GR, APX, MDHAR, DHAR, GST, GPX), and non-enzymatic (ASA, GSH, GSSG) antioxidants got induced by NaCl and *TH* further enhanced the synthesis of these phytoconstituents and protects the *Brassica* plants from further damage. The plants inoculated with *TH* hold potential to induce relative salt tolerance and improve plant growth of Indian mustard under salt stress. By using this sustainable approach we can bring salt affected land under cultivation with appreciable yield.

## Conflict of Interest Statement

The authors declare that the research was conducted in the absence of any commercial or financial relationships that could be construed as a potential conflict of interest.
